# Seasonal variation in the ranging behavior of elephants in the Laikipia‐Samburu ecosystem

**DOI:** 10.1002/ece3.70198

**Published:** 2024-08-27

**Authors:** Loise W. Kuria, Duncan M. Kimuyu, Mwangi J. Kinyanjui, George Wittemyer, Festus W. Ihwagi

**Affiliations:** ^1^ School of Natural Resources and Environmental Studies Karatina University Karatina Kenya; ^2^ Mpala Research Center and Wildlife Foundation Nanyuki Kenya; ^3^ Save the Elephants Nairobi Kenya

**Keywords:** core areas, habitat use, home range shift, home range size, *Loxodonta africana*, mega‐herbivores

## Abstract

African savanna elephants are a highly mobile species that ranges widely across the diversity of ecosystems they inhabit. In xeric environments, elephant movement patterns are largely dictated by the availability of water and suitable forage resources, which can drive strong seasonal changes in their movement behavior. In this study, we analyzed a unique movement dataset from 43 collared elephants, collected over a period of 10 years, to assess the degree to which seasonal changes influences home range size of elephants in the semi‐arid, Laikipia‐Samburu ecosystem of northern Kenya. Auto‐correlated Kernel Density Estimation (AKDE) was used to estimate elephants' seasonal home range size. For each individual elephant, we also calculated seasonal home range shifts, as the distance between wet season home range centroids and dry season home range centroids. Core areas (50% AKDE isopleths) of all individual elephants ranged from 3 to 1743 km^2^ whereas total home range sizes (the 95% AKDE isopleths) ranged between 15 and 10,677 km^2^. Core areas and home range sizes were 67% and 61% larger, respectively, during the wet season than during the dry season. On average, the core area centroids for all elephants were 17 km away from the nearest river (range 0.2–150.3 km). Females had their core areas closer to the river than males (13.5 vs. 27.5 km). Females differed from males in their response to seasonal variation. Specifically, females tended to occupy areas farther from the river during the wet season, while males occupied areas further from the river during the dry season. Our study highlights how elephants adjust their space use seasonally, which can be incorporated into conservation area planning in the face of increased uncertainty in rainfall patterns due to climate change.

## Graphical abstract

This manuscript presents an analysis of the how elephant adjust their space use seasonally. In particular, the paper investigates the variation in home range sizes and home range shifts across seasons. Our results exhibit the importance of tailored conservation strategies that would ensure the longevity of elephant populations while safeguarding landscape connectivity and crucial movement corridors, addressing both immediate and long‐term threats.

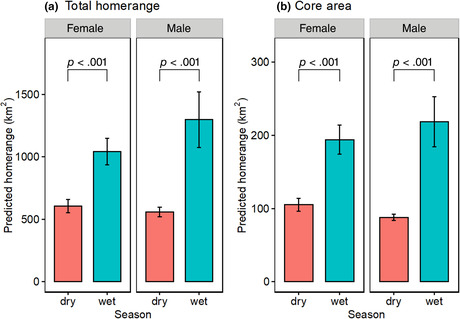

## 1 INTRODUCTION

Elephants act as ecosystem engineers by influencing the structure and complexity of the habitats in which they occur (Gebremeskel Haile et al., 2019), resulting in a myriad of cascading effects on other trophic levels (Calenge et al., 2002; Smallie & O'Connor, 2000). The scale of this effect is relatively broad because elephants are mega‐herbivores with large home ranges and high mobility, allowing them to cover large distances across the landscape as they search for forage resources (Bolla & Hovorka, 2012). As mixed feeders with diverse diets, elephants can exhibit great plasticity in ranging behavior (Bastille‐Rousseau & Wittemyer, 2019; Ortega & Eggert, 2004; Sukumar, 2003). Such changes may be driven by seasonal variation in the availability of forage and water resources (Roever et al., 2012; Sukumar, 2003; Valls‐Fox et al., 2018; Wittemyer et al., 2017) as well as physiological and behavioral differences among individual elephants (Fortin et al., 2022; Moss et al., 2011). Home ranges link the movement of animals to the distribution of the resources necessary for survival and reproduction. Home range size, location, and shape may change depending on the state of the individual and the conditions of the external environment (Börger et al., 2006, 2008; Kenward et al., 2001). There is great interest in examining spatial and temporal variation in elephant‐ranging behavior, but the lack of long‐term longitudinal datasets seriously constrains such studies.

Seasonal pulses in the availability and distribution of forage and water can have enormous impacts on elephants' ranging behavior (Bastille‐Rousseau et al., 2020). Generally, forage and water tend to be more widely available during wet than dry seasons. For example, rainfall creates temporary pools of water that may be utilized by animals, negating the need to rely on more permanent water sources such as rivers and springs. Similarly, an increase in perceived forage availability during the wet season may allow animals to utilize some areas that are generally avoided during the dry season. In response to seasonal fluctuations in resource availability, elephants may either migrate out of an area or exhibit seasonal range fidelity. Migration, defined as the repeated seasonal movement between two non‐overlapping regions (Dingle & Drake, 2007), allows elephants to escape severe seasonal decline in resources. Elephants may employ an extensive continuum of movement behaviors that includes migration, highly variable home ranges, or resident behavior (Bartlam‐Brooks et al., 2011; Purdon et al., 2018). Seasonal range fidelity occurs when an individual changes the size of its range while maintaining the core area of habitat use, consequently presenting relatively high range fidelity but with a change in the degree of range overlap (Damuth, 1981; Lindstedt et al., 1986). Site fidelity is attributable to predictable access to resources (Burton‐Roberts et al., 2022).

Male and female elephants ranging behavior varies across space and time due to their social organization (Fortin et al., 2022; Moss et al., 2011; Wittemyer, Douglas‐Hamilton, et al., 2005) as well as the difference in foraging strategy (Duffy et al., 2011; Kioko et al., 2020; Lee et al., 2011; Woolley et al., 2009). Male elephants may have larger home ranges than females as they disperse to unfamiliar habitats to seek food and mates (du Toit & Moe, 2014; Lee et al., 2011). Unlike male elephants, female African elephants live in matrilineal families consisting of individuals of different ages. Family‐ranging behavior may be constrained by calves that may not be able to move fast and far from water sources (Ngene, 2010). Male elephants, however, move and forage alone or in bachelor herds without calves that would limit their movement (Ngene, 2010).

Elephants in the Laikipia‐Samburu ecosystem are known to move over large areas over time because the ecosystem is strongly seasonal (Bastille‐Rousseau et al., 2020; Wittemyer et al., 2008). In light of this, there was a critical need to document and compare elephant movements over these large areas as one might expect their ranging behavior to vary seasonally. To understand variations in distribution patterns and space use of elephants in this resource‐limiting region, rigorous field data modeled with ecologically meaningful predictor variables is needed. Understanding and predicting patterns of animal space use is particularly important for heavily managed animal populations and for species that may severely affect ecological processes (Reinecke et al., 2014).

To gain a deeper understanding of drivers of elephant space use, we analyzed the movement dataset from 43 collared elephants, collected over 10 years in the Laikipia‐Samburu ecosystem in northern Kenya. Our analysis aimed to assess (i) the variation in home range sizes across seasons and (ii) the extent of seasonal home range shifts of elephants in relation to their proximity to the river. We predicted that elephants would have a larger home range during the wet season to capitalize on dispersed resources. We expected that male elephants would have larger home range sizes than females due to their movement in search of mates and dispersed resources. In contrast, because female elephants live in matrilineal families with dependent calves, they would be constricted to areas near rivers and water sources and expected to have smaller ranges than males. We expected elephants to move from one location to another depending on seasonal resources (primary water) and, therefore, predicted that the elephants' home range would shift to areas closer to the Ewaso Ng'iro river, which is a key perennial water resource (Barkham & Rainy, 1976) during the dry seasons in the ecosystem. We discuss the implications of our results for elephant conservation.

## 2 METHODS

### 2.1 Study area

**FIGURE 1:**
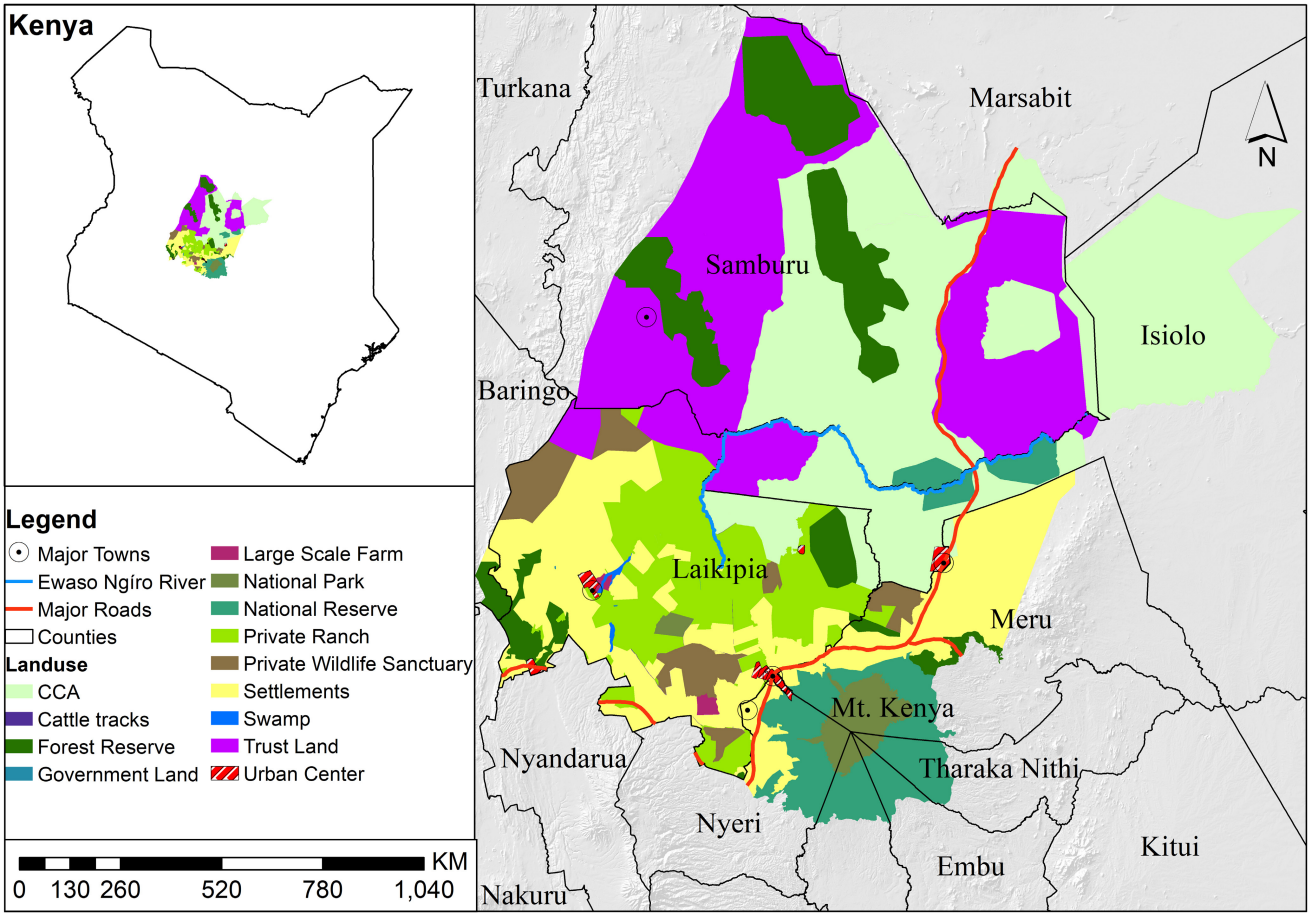
The location of the Laikipia‐Samburu ecosystem in Northern Kenya.

This study was conducted in the Laikipia‐Samburu ecosystem of Kenya using data for 2001 and 2021 (Figure 1). The ecosystem is bounded by coordinates 0.2° S to 1.5° N and 36.2° E to 38° E. The ecosystem is defined by the geographic extent of the Ewaso Nyiro River and its tributaries, encompassing approximately 36,790 km^2^ (Thouless, 1995) and the historical elephant migration range (Georgiadis, 2011). The study area is semi‐arid with a wide range of habitats linked with the elevation and climatic gradients that characterize the region: from cool, wet highlands in the south to hot, dry lowlands in the north (Georgiadis, 2011). The rainfall is highly variable and bimodal, with peaks in May and November and a yearly range from <400 mm in the north to a maximum of 600 mm in the south (Barkham & Rainy, 1976; Ihwagi et al., 2012). The terrain is comprised of expansive plains interrupted by rugged terrain and isolated hills. Wildlife shares the landscape freely with the predominantly pastoral communities (Ihwagi et al., 2015). The confirmed Laikipia‐Samburu elephant range encompasses six major land use types: community conservancies, private ranches, communal pastoral areas, state‐protected forest reserves, settlements mainly under sedentary subsistence production, and the national reserves that are either owned by individuals, government, or communities. The private, government, and community lands comprise 30%, 11%, and 59% of the landscape, respectively. The Laikipia–Samburu elephant (*Loxodonta africana*) population is the second largest in Kenya, with approximately 7347 individuals, primarily relying on the range outside of governmentally protected areas (Litoroh et al., 2010).

### 2.2 Data collection

#### 2.2.1 Elephant movement behavior

**FIGURE 2:**
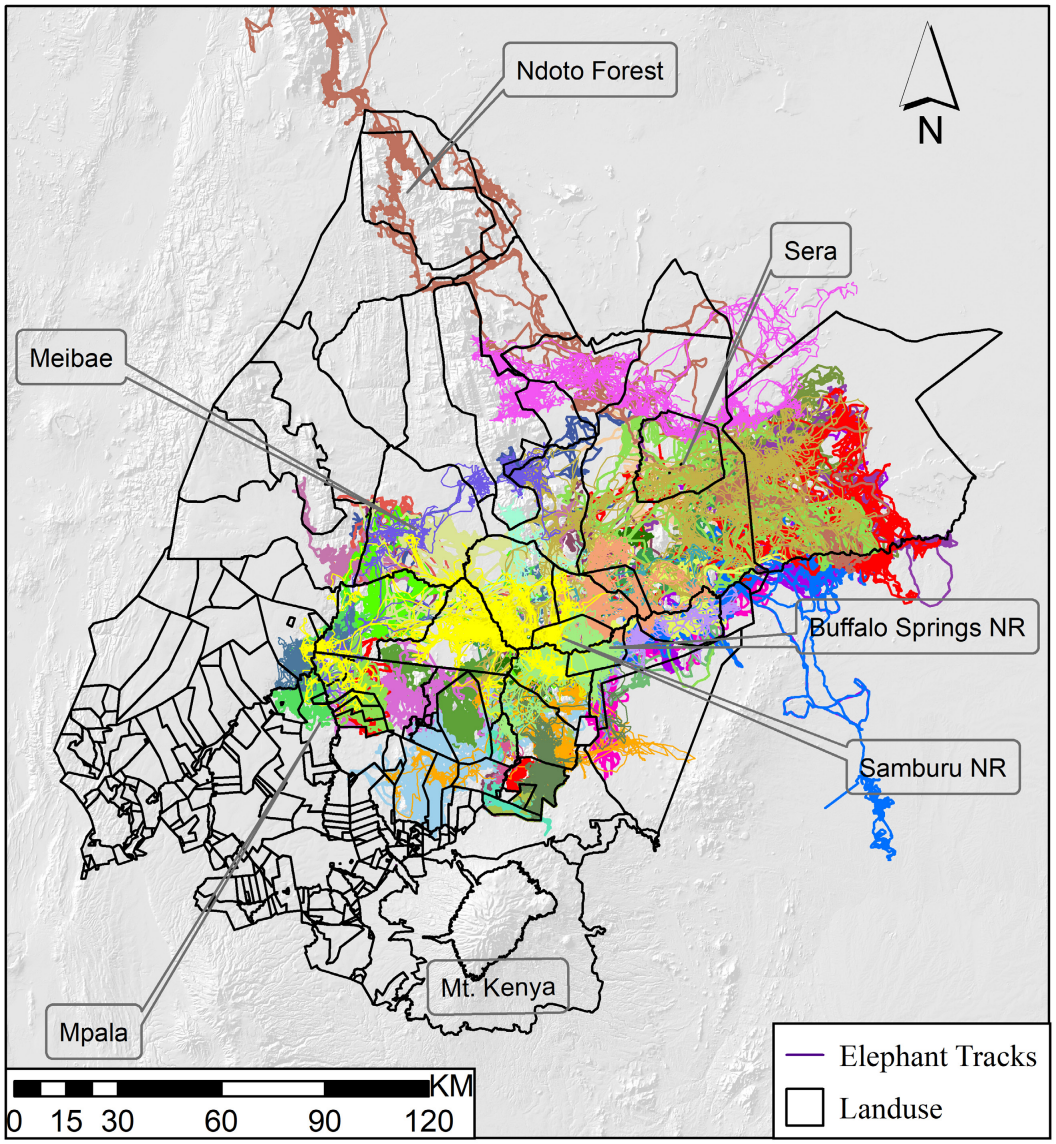
Range extent of the 43 elephants (each represented by a random color) tracked in the Laikipia‐Samburu ecosystem.

Existing GPS‐satellite data from 43 collared elephants (14 males and 29 females) that use the Laikipia‐Samburu ecosystem were analyzed (Figure 2). Male African elephants are solitary, whereas female African elephants live in matrilineal families that can have as many as 36 members (Wittemyer, 2001; Wittemyer, Daballen, et al., 2005; Wittemyer, Douglas‐Hamilton, et al., 2005). Only one individual elephant was tracked for family groups consisting of multiple females. Save the Elephants carried out elephant collaring operations with Kenya Wildlife Service using standard operating procedures. These collars came from either African Wildlife Tracking from South Africa, Savannah Tracking from Kenya, or Followit AB from Sweden. Cost influences the collar used, the environment they are deployed in, and technical specifications. The collars recorded the location of each elephant at a set of 1‐h intervals. Elephant tracking data were retrieved from a centralized database using customized software that employs a data filter to remove erroneous GPS fixes based on a maximum rate of travel of 7 km/h (Wall et al., 2014). The data projections were on the Universal Transverse Mercator (UTM) WGS‐84 reference system zone 37 N. Data were stored in ESRI Geodatabase (ArcGIS version 10). Data collected between 2001 and 2021 were used for this study. All 43 elephants that were selected for this study had been tracked at various intervals during this period, but each elephant had at least 1 year of continuous tracking data with at least 10 months of tracking data in any given year. The average tracking period for all 43 individuals was 4.3 years (range 1–9) years, with the most tracked between 2015 and 2018 (Table S1).

#### 2.2.2 Seasons data delineation

**FIGURE 3:**
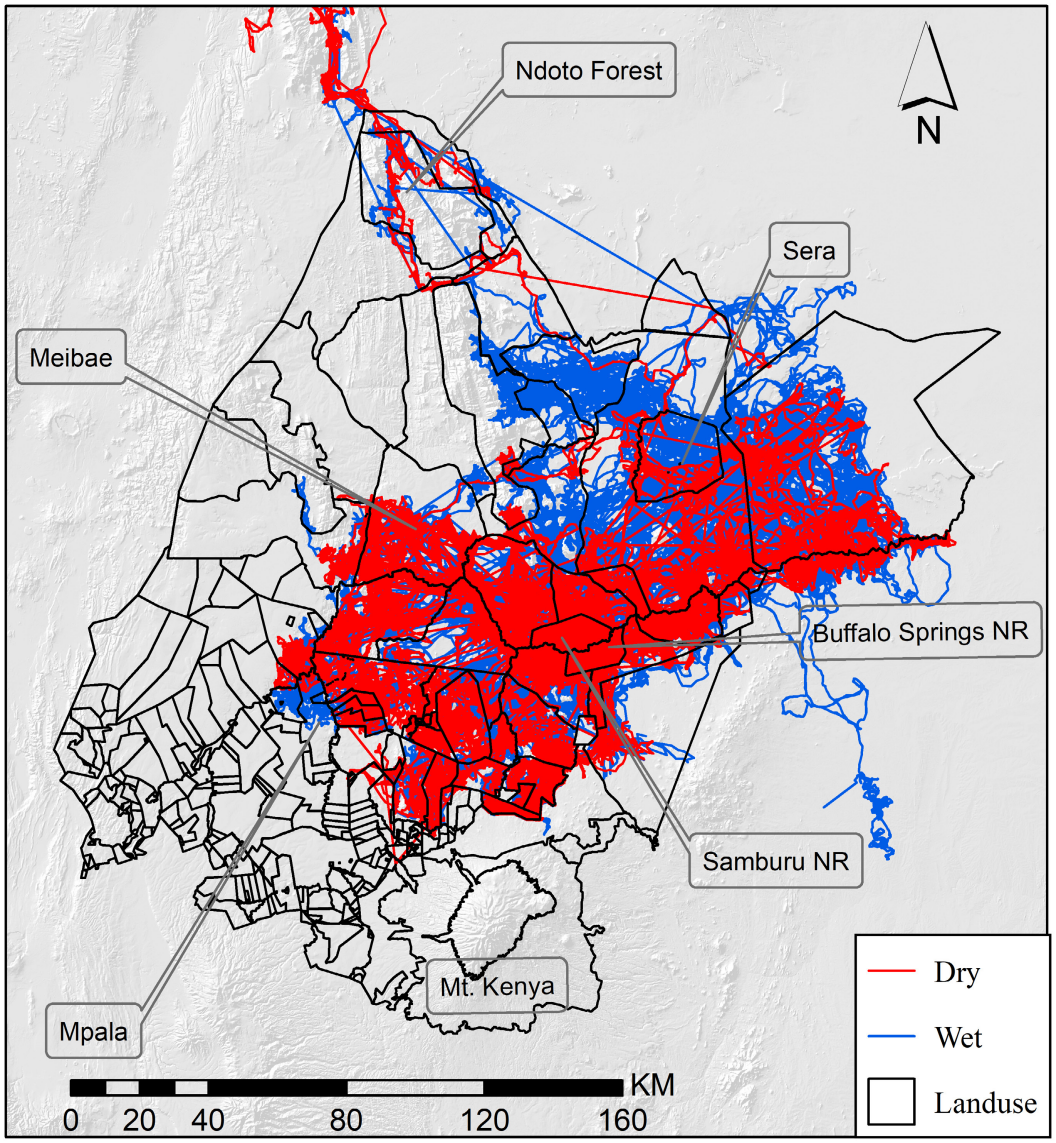
Range extent of elephants during the wet and dry seasons from GPS tracking data collected from 43 individuals in the Laikipia‐Samburu ecosystem.

Ecoscope (Copyright 2022, Wildlife Dynamics, https://ecoscope.io/) an open‐source Python library used for environmental and conservation data analyses was used to identify transitions between wet and dry seasons. Using std_ndvi_vals function in the Ecoscope tool, we extracted standardized NDVI (Normalized Difference Vegetation Index) values within the study area. NDVI values range between −1 and 1; with values closer to 1 representing higher productivity (i.e., wet seasons) and values closer to −1 representing lower productivity (i.e., dry seasons). NDVI reflects actual changes in vegetation attributes, therefore, it may be considered a better proxy for seasonal changes, than rainfall data. The NDVI values were extracted for the whole study period (2001–2021). The *val_cuts* function was used to calculate the seasonal transition point, which is the point where the NDVI values change from increasing to decreasing or vice versa. The seasonal windows function was then used to determine the seasonal time windows. The output was a data frame containing each season's start and end dates, along with a label for each season. The seasonal time windows data frame was exported to a CSV file. The season's data were used to determine whether an elephant location fix was during the wet or dry seasons, hence splitting the elephant movement data into two (Figure 3). This was achieved by assigning an elephant location GPS fix time to either wet or dry depending on the season.

### 2.3 Data analyses

The Autocorrelated Kernel Density Estimation (AKDE) method was used to estimate the individual elephants' core area (areas bounded by 50% isopleths) and total home range size (areas bounded by 95% isopleths) for every wet and dry instance in a year. We considered any period lasting more than 80 days of continuous wet or dry periods as a ‘significant’ wet or dry instance. Wet seasons in the area typically last 80 days, and the area experiences two wet seasons in a year. Therefore, an average year would have two wet and two dry instances. AKDE was considered appropriate because it was designed to be statistically efficient when dealing with the complexities and biases of modern movement data, such as autocorrelation and missing data (Silva et al., 2022). Semi‐variance functions (SVF) were calculated for each elephant to assess for range residency in all datasets (Calabrese et al., 2016). Range residency is an assumption of the AKDE approach and is necessary for the method to define a home range accurately. The elephants used in this study are residents of the Samburu Laikipia ecosystem. We calculated the Weighted AKDEc (or wAKDEc) to cater for when the device had a malfunction, leading to GPS fixes shifting from one fix per hour to one fix per ‘*n*’ hours. We estimated the seasonal home range shift in relation to their proximity to rivers by calculating the displacement of the centroids of individual elephant core areas during the wet and dry seasons. We used ArcGIS (Esri, 2011) to compute the core area centroids, which were calculated using the centre of gravity algorithm (Bartlett et al., 2016). The centroids' coordinates were then extracted and used to determine the shift in the individual elephant core areas across seasons. ArcGIS was also used to calculate the distance from the centroids to the river. This analysis allowed us to quantify and compare the movement patterns of elephants between the dry and wet seasons, providing insights into how their core areas change in response to seasonal changes.

Generalized linear mixed models (GLMMs) with Gamma error structure and log link function were then used to test the effect of season and sex of individual elephants on both the total home range and core areas. All our models included elephant ID as a random effect to account for repeated sampling of individuals' (multiple tracking instances). To account for differences in sampling effort (differences in the number of days per tracking instance), we have included sampling days per instance as an offset in our models. We fitted all our models using the generalized linear mixed effect model ‘glmer’ function of the ‘lme4’ package (Bates, 2010; Bates et al., 2015). Generalized linear model (GLM) was used to test the effect of season and sex on seasonal home range shift in relation to their proximity to river Ewaso Ngi'ro. Similarly, to account for differences in sampling effort, we have included sampling days per instance as an offset in our models. Akaike's Information Criterion (AIC) Weighting was used for model averaging and to evaluate support for competing ranging behavior models. Model selection and averaging were performed using the R package MuMIn version 4.2.0 (Barton, 2016). All statistical analyses were performed using R (R Core Team, 2021), and all tests were carried out at a significance level of 0.05.

## 3 RESULTS

**FIGURE 4:**
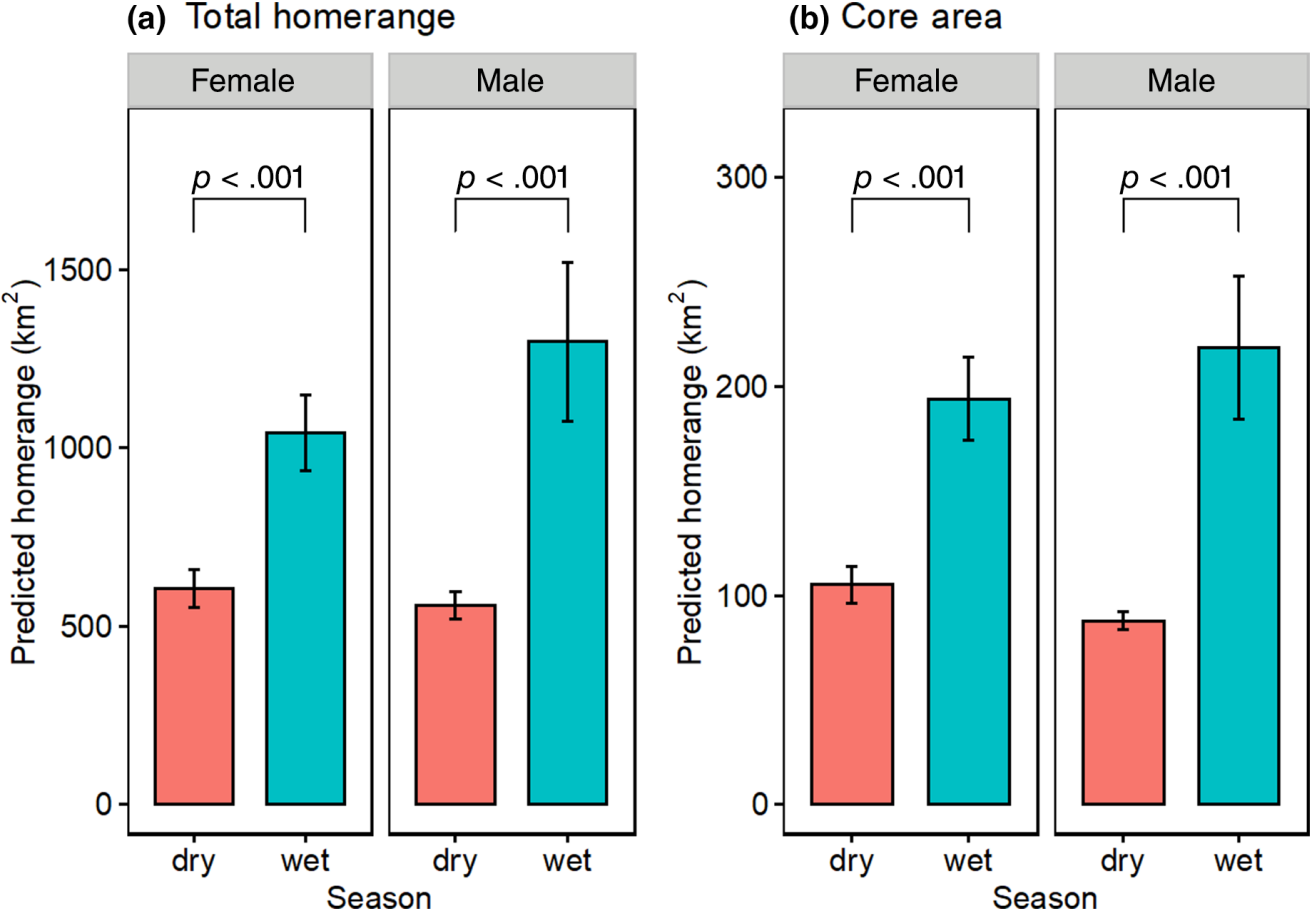
Seasonal differences in predicted (a) total home range and (b) core areas of elephants in Laikipia‐Samburu ecosystem. Predicted home range values are obtained from our best‐candidate model. Error bars represent standard errors (± one SE).

A total of 771,919 hourly GPS fixes were collected during the dry season and 990,772 during the wet season. These data represented 340 individual seasons across 2001–2021. All individual elephants' core areas ranged between 3 and 1743 km^2^, whereas the total home range ranged between 15 and 10,677 km^2^. Based on AICc values, the best ranking models included season only, and season and sex as the best predictors of total home range size, as well as core areas (Table 1). We considered the later model (including season and sex) because it aligns better with theoretical expectations. On average, the core (50% isopeth) home range size was 67% (101 km^2^) larger during wet than the dry season (*χ* ^2^ = 21.06, *p* < .001). Similarly, the total home range size was 61% (521 km^2^) larger during wet than the dry season (*χ* ^2^ = 15.45, *p* < .001). Sex did not influence the individual elephant's core area and home range size (*χ* ^2^ = 0.63, *p* = .429; *χ* ^2^ = 1.07, *p* = .300 respectively, Figure 4).

**TABLE 1:** Candidate models of the temporal variation of the elephant ranging behavior in relation to the season in the Laikipia‐Samburu ecosystem, Kenya, 2001–2021.

	Model	AICc	Delta
Total home range size	Season	5089.15	0
	Sex + season	5090.14	0.99
	Season * sex	5092.17	3.027
	1	5101.97	12.819
	Sex	5102.95	13.802
Core area size	Season	3918.16	0
	Sex + season	3919.59	1.433
	Season * sex	3921.45	3.291
	1	3936.21	18.055
	Sex	3937.63	19.476
Core area shift	Sex * season	2195.84	0
	Sex	2197.95	2.119
	Sex + season	2200	4.168
	1	2226.39	30.55
	Season	2227.12	31.29

*Note*: All models included elephant identification as a random factor and the number of days per tracking instance as an offset.

**FIGURE 5:**
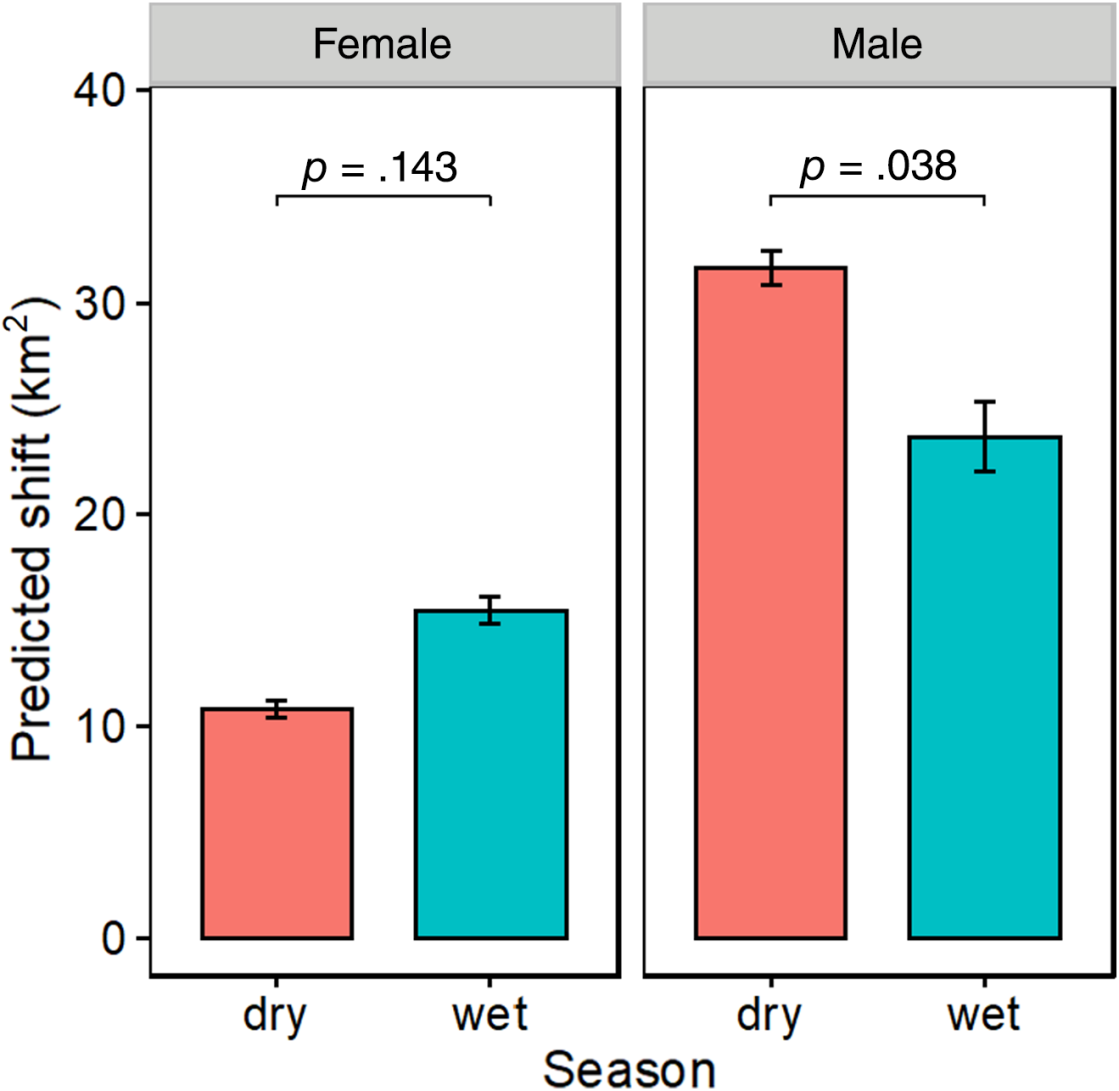
Seasonal differences in predicted core area shift of elephants in Laikipia‐Samburu ecosystem. Predicted core area values are obtained from our best candidate model. Error bars represent standard errors (± one SE).

On average, the core area centroids for all elephants were 17 km away from the nearest river (range 0.2–150.3 km). Females had their core areas closer to the river than males (13.5 vs. 27.5 km). Based on AICc values, the best ranking model included season and sex as the best predictor of home range shift (Table 1). Females differed from males in their response to seasonal variation (*χ* ^2^ = 6.43, *p* = .011). Females tended to occupy areas farther from the river during the wet season, while males occupied areas further from the river during the dry season (Figure 5).

## 4 DISCUSSION

Understanding elephant ranging behavior in arid environments can provide insight into ecological constraints and requirements for the species. We had predicted that elephants would have larger home ranges and core areas during the wet season than during the dry season, and that males would have overall larger ranges than females. We found evidence in support of our first prediction; but not that males have larger home ranges than females. Our prediction that elephants would shift their home range based on seasons was supported. Lastly, our prediction that elephants would shift their core areas to occupy areas proximate to the river (perennial water source) during the dry season was partially supported.

The differences between wet and dry season core areas and the home range size observed in this study can be attributed to increased dispersion of water and forage resources during the wet season, allowing elephants to utilize sections of the habitat that are normally avoided during the dry season. In our study, the river Ewaso Ng'iro is the only water source during the dry season. However, numerous ephemeral water pools become available during the wet season (Ihwagi et al., 2010) resulting in high‐quality forage resources becoming more available away from the river, implying that elephants can take long forays away from the river. Our findings concur with several other studies that have shown that elephants tend to reduce their home ranges during the dry season by concentrating their foraging activities in areas close to water (ChamaillÉ‐Jammes et al., 2007; de Beer et al., 2006; Leggett, 2006; Osborn & Parker, 2003; Owen‐Smith, 2004; Redfern et al., 2003; Smit et al., 2007). The effect of seasonal shrinking and expansion of elephant home range warrants subsequent investigation. One possibility is that reduction in home range size during the dry season may concentrate elephant browsing damage to small‐isolated patches, significantly impacting vegetation. However, such patches may recover from heavy browsing once elephants disperse during the wet season.

The observed increase in home range and core area size during the wet season was consistent for both male and female elephants. These findings concur with an earlier study that showed that the home range of sexually immature males, non‐musth, sexually mature males, and females is similar (Whitehouse & Schoeman, 2003). However, sexually active males have been reported to have more extensive home ranges than females (Barnes, 1983; Leggett, 2006; Poole & Moss, 1989; Taylor et al., 2020; Viljoen, 1989; Whitehouse & Schoeman, 2003). In our study, we did not account for the reproductive status of individual elephants. However, we expect such similarities could be mooted because the Samburu‐Laikipia ecosystem experiences long dry periods, and both males' and females' movements are limited to areas near water points during the dry season.

This study showed that on average, females tended to occupy areas further from the river during the wet season, while males occupied areas further from the river during the dry season. This is because during the dry seasons the resources available, that is, water and food, become limited. Therefore, for the free roaming adult male elephants must move away from the river in search of food. In the wet season, male elephants prefer to stay close to the river. This is because of the availability water and high quality forage near the river during the wet season with the consistent need to drink water as elephant require drinking water every one or 2 days (Douglas‐Hamilton, 1973). Shannon concurred with the results as she noted that elephants are attracted to habitats near rivers and dams, which not only provide drinking water but also an abundance of forage (Shannon et al., 2006). A study done by Leggett found similar results with distinct seasonal movement of collared elephants between their wet and dry season core areas (Leggett, 2006). However, Viljoen reported that the elephants of the northwest restrict themselves to seasonal ranges within their individual home ranges, irrespective of higher rainfall or river floods in areas adjacent to their home ranges (Viljoen, 1989). During the dry season, female elephants are more likely to shift their core area to occupy areas near the river, where water resources are more available because female elephants' movement is restricted by dependent calves. This explains why females had their core areas closer to the river than males. This seasonal behavior underscores the importance of rivers and water sources for the Samburu‐Laikipia ecosystem elephants' survival and movement patterns.

The AKDE home ranges observed for GPS‐collared elephants in the Laikipia‐Samburu ecosystem ranged from 22 to 10,677 km^2^. The home range sizes of this study are comparable to home ranges reported in other studies (Table 2). The maximum area of the total home range for the female reported in this study is 10,677 km^2^ while the total home range for males in this study is 6921 km^2^. The large home ranges are comparable to the desert‐dwelling elephants of Mali with an approximate home range of 11,500–23,980 km^2^ (Wall et al., 2013) as they had larger home ranges than previously reported for savanna elephants. The larger home ranges of elephants in northwest Namibia probably reflect the type and quality of available vegetation (Viljoen, 1989). The large home range sizes reported in this study could be because of the unpredictable availability of water. Secondly, food availability may be limited, thus forcing animals to move over a wider area and/or the spatial distribution of habitat types of higher nutritional quality may result in a wider area being traversed. The home range sizes determined by radio‐tracking collars were usually larger than the ones revealed by visual identification (Leuthold, 1977). The small home range size of some elephants could be because of physical barriers therefore confining them to a smaller range of habitat. The home range sizes of 14 to 52 km^2^ for the African elephant at Lake Manyara National Park, Tanzania (Douglas‐Hamilton, 1972) could be imposed by the dense vegetation in the park, likely providing a high density of forage for elephants.

**TABLE 2:** Autocorrelated kernel density estimation (AKDE) home range estimates of elephants from Bukit Tigapuluh, Khaudum, Etosha, Zambezi, and Kunene.

Region	Home range size (km^2^)	References
Bukit Tigapuluh, Indonesia	275–5180	Moßbrucker et al. (2016))
Khaudum National Park, Namibia	3000–12,000	Benitez et al. (2022)
Etosha National Park, Namibia	280–38,000	Benitez et al. (2022)
Zambezi, Namibia	1400–25,000	Benitez et al. (2022)
Kunene region of northwestern Namibia	1500–37,000	Benitez et al. (2022)

Samburu‐Laikipia ecosystem is among the many ecosystems worldwide that are facing increased fragmentation. Factors contributing to such fragmentation include intensive agriculture, overgrazing, conversion of large areas to human settlements, illegal logging, urbanization, etc. Such fragmentation will likely affect the distribution patterns of elephants and other herbivores in these systems. The future climatic projections suggest an increase in drought frequency and severity in the region (Gebremeskel Haile et al., 2019). Such changes may have severe implications for elephants as well as other biodiversity. Elephants, among other species, contract their range to remain near permanent water sources. Spending more time in the vicinity of water intensifies the browsing pressure in those regions and may cause remarkable changes in vegetation cover and composition. In the Laikipia‐Samburu ecosystem, Ewaso Ng'iro River tends to retain water for a prolonged period during drought, attracting animals from different regions to the area. Conservation efforts should focus on maintaining a network of conservation areas that is large enough to accommodate seasonal expansion in home range or migration of animals while still maintaining access to critical resources such as water and dry season foraging habitats.

In conclusion, the behavioral patterns of elephants in the Samburu‐Laikipia ecosystem are intricately tied to seasonal variations. The wet season prompts the expansion of home range as water and food resources become more abundant across the landscape. Contrary to our predictions, male elephants did not exhibit significantly larger home ranges than females, indicating that females as well as males demonstrate the use of vast areas. The prolonged dry periods in the Laikipia‐Samburu ecosystem facilitate elephant home range shifts across seasons. This raises questions about potential trade‐offs between foraging near watering points with lower‐quality resources and embarking on costly journeys to distant food patches. Given these challenges, the Laikipia‐Samburu ecosystem necessitates tailored conservation strategies such as protection and management of movement corridors, community engagement and education, habitat restoration among others that would ensure the longevity of elephant populations while safeguarding landscape connectivity and crucial movement corridors, addressing both immediate and long‐term threats.

## AUTHOR CONTRIBUTIONS

**Loise W. Kuria:** Conceptualization (equal); data curation (lead); formal analysis (lead); methodology (equal); writing – original draft (lead); writing – review and editing (equal). **Duncan M. Kimuyu:** Conceptualization (equal); data curation (equal); formal analysis (equal); methodology (equal); writing – review and editing (equal). **Mwangi J. Kinyanjui:** Funding acquisition (supporting); writing – review and editing (supporting). **George Wittemyer:** Writing – review and editing (equal). **Festus W. Ihwagi:** Conceptualization (equal); data curation (equal); formal analysis (equal); funding acquisition (lead); methodology (equal); writing – review and editing (equal).

## CONFLICT OF INTEREST STATEMENT

The corresponding author confirms on behalf of all authors that no involvements might raise the question of bias in work reported or in the conclusions, implications, or opinions stated.

## Supporting information


Table S1.

## Data Availability

The data on home range size and shift across seasons are publicly available on the Dryad website (https://doi.org/10.5061/dryad.djh9w0w77). GPS positions of elephant distribution are ethically restricted as the locations may jeopardize the anti‐poaching operations and thus put elephants at greater risk. The data are however available on written request from the Office of Senior Assistant Director of Kenya Wildlife Service.
