# Supplementary material for: Seasonal variation in the ranging behavior of elephants in the Laikipia‐Samburu ecosystem
Source: Ecol Evol. 2024 Aug 27;14(8):e70198. doi: 10.1002/ece3.70198 (PMC11347863; doi:10.1002/ece3.70198)
Supplement: Supplementary file 1 — Table S1. [file ECE3-14-e70198-s001.docx]

# **Supporting information**

## Table S1: Tracking period of individual elephants in the Laikipia-Samburu ecosystem.

| Individual ID | 2001 | 2002 | 2003 | 2008 | 2009 | 2010 | 2011 | 2012 | 2013 | 2014 | 2015 | 2016 | 2017 | 2018 | 2019 | 2020 | 2021 | Duration (Years) |
| --- | --- | --- | --- | --- | --- | --- | --- | --- | --- | --- | --- | --- | --- | --- | --- | --- | --- | --- |
| Abdi Boru |  |  |  |  |  |  |  |  |  |  |  |  |  |  |  |  |  | 5 |
| Amity |  |  |  |  |  |  |  |  |  |  |  |  |  |  |  |  |  | 6 |
| Annabelle |  |  |  |  |  |  |  |  |  |  |  |  |  |  |  |  |  | 9 |
| Arden |  |  |  |  |  |  |  |  |  |  |  |  |  |  |  |  |  | 2 |
| Betsy |  |  |  |  |  |  |  |  |  |  |  |  |  |  |  |  |  | 4 |
| Chukwi |  |  |  |  |  |  |  |  |  |  |  |  |  |  |  |  |  | 2 |
| Drachmae |  |  |  |  |  |  |  |  |  |  |  |  |  |  |  |  |  | 7 |
| Edison |  |  |  |  |  |  |  |  |  |  |  |  |  |  |  |  |  | 7 |
| Eyasi |  |  |  |  |  |  |  |  |  |  |  |  |  |  |  |  |  | 4 |
| Femur |  |  |  |  |  |  |  |  |  |  |  |  |  |  |  |  |  | 2 |
| Flaubert |  |  |  |  |  |  |  |  |  |  |  |  |  |  |  |  |  | 4 |
| Frank |  |  |  |  |  |  |  |  |  |  |  |  |  |  |  |  |  | 2 |
| Gambela |  |  |  |  |  |  |  |  |  |  |  |  |  |  |  |  |  | 5 |
| Habiba |  |  |  |  |  |  |  |  |  |  |  |  |  |  |  |  |  | 8 |
| Jangfrau |  |  |  |  |  |  |  |  |  |  |  |  |  |  |  |  |  | 4 |
| Jerusalem |  |  |  |  |  |  |  |  |  |  |  |  |  |  |  |  |  | 2 |
| Jordan |  |  |  |  |  |  |  |  |  |  |  |  |  |  |  |  |  | 4 |
| Kiir |  |  |  |  |  |  |  |  |  |  |  |  |  |  |  |  |  | 3 |
| Kili |  |  |  |  |  |  |  |  |  |  |  |  |  |  |  |  |  | 5 |
| Kimita |  |  |  |  |  |  |  |  |  |  |  |  |  |  |  |  |  | 4 |
| Laresoro |  |  |  |  |  |  |  |  |  |  |  |  |  |  |  |  |  | 5 |
| Learata |  |  |  |  |  |  |  |  |  |  |  |  |  |  |  |  |  | 4 |
| Leparua |  |  |  |  |  |  |  |  |  |  |  |  |  |  |  |  |  | 6 |
| Loibor |  |  |  |  |  |  |  |  |  |  |  |  |  |  |  |  |  | 3 |
| Luna |  |  |  |  |  |  |  |  |  |  |  |  |  |  |  |  |  | 6 |
| Madurba |  |  |  |  |  |  |  |  |  |  |  |  |  |  |  |  |  | 2 |
| Mbalambala |  |  |  |  |  |  |  |  |  |  |  |  |  |  |  |  |  | 4 |
| Miguna Miguna |  |  |  |  |  |  |  |  |  |  |  |  |  |  |  |  |  | 2 |
| Naisula |  |  |  |  |  |  |  |  |  |  |  |  |  |  |  |  |  | 5 |
| Ntepes |  |  |  |  |  |  |  |  |  |  |  |  |  |  |  |  |  | 5 |
| Nutmeg |  |  |  |  |  |  |  |  |  |  |  |  |  |  |  |  |  | 5 |
| Nyiro |  |  |  |  |  |  |  |  |  |  |  |  |  |  |  |  |  | 4 |
| Parchuma |  |  |  |  |  |  |  |  |  |  |  |  |  |  |  |  |  | 4 |
| Salma |  |  |  |  |  |  |  |  |  |  |  |  |  |  |  |  |  | 4 |
| Serteta |  |  |  |  |  |  |  |  |  |  |  |  |  |  |  |  |  | 3 |
| Shafaa |  |  |  |  |  |  |  |  |  |  |  |  |  |  |  |  |  | 6 |
| Stratus |  |  |  |  |  |  |  |  |  |  |  |  |  |  |  |  |  | 3 |
| Sue |  |  |  |  |  |  |  |  |  |  |  |  |  |  |  |  |  | 2 |
| Tassia |  |  |  |  |  |  |  |  |  |  |  |  |  |  |  |  |  | 5 |
| Taurus |  |  |  |  |  |  |  |  |  |  |  |  |  |  |  |  |  | 8 |
| Teresai |  |  |  |  |  |  |  |  |  |  |  |  |  |  |  |  |  | 3 |
| Tia Maria |  |  |  |  |  |  |  |  |  |  |  |  |  |  |  |  |  | 5 |
| Timurid |  |  |  |  |  |  |  |  |  |  |  |  |  |  |  |  |  | 4 |
| Yale |  |  |  |  |  |  |  |  |  |  |  |  |  |  |  |  |  | 4 |

## 
